# Alpha-Glycerylphosphorylcholine and D-Panthenol Eye Drops in Patients Undergoing Cataract Surgery

**DOI:** 10.1155/2022/1951014

**Published:** 2022-06-07

**Authors:** Gianluca Martone, Alessandra Balestrazzi, Giorgio Ciprandi, Angelo Balestrazzi

**Affiliations:** ^1^UOC Oculistica, Usl Sud-Est Toscana, Grosseto, Italy; ^2^UOC Oculistica, ASL ROMA 2, Rome, Italy; ^3^Allergy Clinic, Casa di Cura Villa Montallegro, Genoa, Italy

## Abstract

Cataract surgery is widespread. The surgical procedure is associated with damage to the epithelial barrier and interruption of the corneal innervation. In addition, pathological events sustain signs and symptoms that may persist for a long time. Recently, a fixed combination of alpha-glycerylphosphorylcholine and D-Panthenol (Oftassiale) has been available as eye drops. The present study investigated the effects of an Oftassiale therapy in 20 patients undergoing cataract surgery. A comparison group included 20 patients treated with topical hyaluronic acid. Standard prophylactic and anti-inflammatory treatment was prescribed to all patients. Clinical signs and symptoms were assessed over time. In vivo confocal microscopy (IVCM) was performed accordingly. Oftassiale treatment significantly reduced clinical features and improved IVCM outcomes. In addition, therapy was well-tolerated, and no clinically significant adverse events occurred. In conclusion, this study confirmed that IVCM helps assess the tunnel after cataract surgery due to its ability to provide microscopic details in vivo. Topical therapy with alpha-glycerylphosphorylcholine and D-Panthenol eye drops promoted and stabilized the reepithelialization process. This fixed combination also accelerated and modulated the repair of the corneal innervation. Moreover, this treatment was well-tolerated and safe.

## 1. Introduction

Cataract is an ocular condition that causes vision impairment due to changes in the ocular lens, leading to a loss of optical clarity and blurred vision. The only cure for cataract is a surgical procedure: the opaque lens is removed by phacoemulsification technique and replaced with an artificial intraocular lens (IOL).

Recovery after phacoemulsification cataract surgery is commonly uncomplicated; however, there is the appearance of complaints after surgery [1]. Surgical care for cataract includes exposure to the microscope's light and perioperative use of ocular drugs that may produce ocular surface stress and irritation. Any extracellular stress affects tear fluid composition and, consequently, the secretion of pro-inflammatory mediators [2]. However, the corneal cut represents the primary stress for the eyes during cataract surgery. Cataract surgery requires a corneal incision, a procedure that induces trauma in the corneal epithelial cells and causes damage to the corneal nerves. The surgical ocular trauma triggers the inflammatory cascade where the cleavage of arachidonic acid by phospholipase-2 produces leukotrienes, prostaglandins, and thromboxanes [3].

Moreover, the onset or aggravation of dry signs and symptoms of dry eye after phacoemulsification, including corneal epitheliopathy and alteration of ocular surface homeostasis, can result in tear film instability, hyperosmolarity, and inflammation. Many studies investigated the changes in tear film stability after cataract surgery using fluorescein break-up time (F-BUT) and the Schirmer test. The effect of phacoemulsification showed a postoperative decrease in F-BUT and Schirmer values, and this effect tends to run out after three months [4].

Recently, an ophthalmic fixed combination has been launched, including alpha-GPC and D-Panthenol (Oftassiale, DMG, Italy). Based on this background, the present study aimed to evaluate the effect of alpha-glycerylphosorylcholine/D-Panthenol eye drops in patients undergoing cataract surgery.

In vivo confocal microscopy (IVCM), a noninvasive ophthalmic imaging diagnostic technique, can provide high-resolution images at the cellular level to show the structural features of the ocular surface tissue.

Corneal nerves have a pivotal role in regulating corneal sensation, maintaining epithelial integrity and proliferation, and promoting wound healing. ICVM offers a unique method to study corneal nerve alterations without altering the tissue microenvironment [5–7].

The present study compared the clinical and morphological results acquired through IVCM between patients undergoing topical alpha-glycerylphosphorylcholine and D-Panthenol solution (Oftassiale) and a control group of patients treated with topical sodium hyaluronate to demonstrate the effects at the level of the corneal incision after cataract surgery.

## 2. Materials and Methods

This prospective, randomized, blinded study was conducted on 40 eyes of 40 patients undergoing standard phacoemulsification surgery with folding IOL implantation in the capsular bag at the Ophthalmology Unit of the University of Siena. Written informed consent to enrollment was obtained from all patients before the study.

Inclusion criteria included patients undergoing phacoemulsification with IOL implantation in the injector bag. Exclusion criteria included surgery with incisions greater than 2.2 mm or intraoperative complications. Patients with corneal dystrophy, preoperative ocular surface disease, previous history of recent ocular inflammation or intraocular surgery, history of uveitis, autoimmune disease, diabetes, glaucoma, use of topical antiglaucomatous or other ocular therapy, a systemic treatment known to affect tear secretion, and previous use of contact lenses were also excluded. In addition, patients who were sutured at the incision level at the end of the operation were excluded.

The same surgeon (A. B.) performed the surgery under topical anesthesia with a self-sealing tunnel in the clear cornea at the temporal level of 2.2 mm. Particular attention was paid to the construction of the engraving. The intervention was a standard phacoemulsification technique with lOL (SN60WF) implantation in the capsular bag using the monarch II injection system with a C-size cartridge (Alcon, Texas, USA). At the end of the surgery, stromal hydration of the principal and lateral incisions was performed to assess the absence of leakage.

All patients used NSAID eye drops three days before surgery and 20 days after surgery, a third-generation quinolone topical antibiotic as prophylaxis for endophthalmitis, for one week after surgery. In addition, a topical antibiotic-steroidal combination was used after surgery for 20 days.

Patients were divided into two homogeneous groups of 20 people. The first group (Group 1) included subjects treated with Oftassiale, administered three times a day from the first postsurgery day, and continued for one month. The second group of patients (Group 2) underwent topical postoperative therapy based on hyaluronate sodium eye drops three times a day for one month. Each patient was evaluated the day before the surgery (baseline), the day after, and on the 30^th^, 60^th^, and 90^th^ postoperative days.

All patients were subjected to clinical examination with a slit lamp, Schirmer test, tear break-up time (TBUT), and examination with IVCM.

Schirmer test was done to test basal and reflex tear secretion using a specialized Schirmer strip marked 0 to 10 mm. Depending on the wetting of the strip, the results of Schirmer test were measured.

TBUT was assessed to test tear film stability and meibomian gland disorder, and the grading was done depending upon the time between the last blink and the appearance of a dry spot.

The confocal laser scanning microscope (HRT II Rostock Corneal Module, Heidelberg Engineering, Germany) uses a 670-nm wavelength diode laser source. The digital images obtained measure 400 *μ*m × 400 *μ*m.

Before and after cataract surgery, the same operator (G. M.) performed IVCM to examine the peripheral cornea temporally at the level of the surgical incision. The examination was performed under topical anesthesia with oxybuprocaine. A drop of gel (Viscotirs Gel; Medivis, Italy) was inserted between the polymethylmethacrylate capsule and the polymethylmethacrylate objective of the confocal microscope and in the lower conjunctival fornix. During the examination, all patients were asked to fix a nasal fixation point to visualize the incision better and minimize optical distortions in the incision area. For each eye examined, at least 20 images of the corneal epithelium, tortuosity, and reflectivity of the subbasal nerve plexus and stroma were obtained. The three most representative images evaluated in a masked way were selected.

The following parameters were considered:

1. *Number of temporal nerve fibers*: this parameter is defined as the sum of the nerve fibers present in an image.
2. *Number of bead-like formations*: this parameter is defined as the number of 1 mm long beading formations of the nerve fiber. Beads are highly reflective bulbous portions of the subbasal nerve. Furthermore, a qualitative morphological analysis evaluated the epithelial, stromal, and endothelial alterations at the incision level.

Results are shown as mean and standard deviation (SD). The *t*-test for unpaired data was used.

## 3. Results

The two groups were comparable in age (71.4 ± 4.9 years in Group 1 and 73.3 ± 5.8 years in Group 2, *P* > 0.05).

After surgery, there was a difference in ocular surface stability between the groups, as evidenced by tests such as TBUT and Schirmer. In Group 1, there was a statistically significant improvement in TBUT compared to the preoperative assessment, which increased from 9.6 ± 2.89 to 11.7 ± 2.24 at one month (*P*=0.02) and 11.1 ± 1.96 at three months (*P*=0.03). However, in Group 2, this improvement in TBUT between preoperative (10.1 ± 2.09) and postoperative values was not observed at 1 (9.8 ± 3.03 *P*=0.73) and at 3 months (10.3 ± 2.44 *P*=0.88). In addition, there was a statistically significant difference between Group 1 and Group 2 at one month (*P*=0.024). Similar improvements were also observed in Group 1 between preoperative (11.8 ± 3.86) and one-month (13.7 ± 2.92 *P*=0.03) and 3-month (13.6 ± 2.45 *P*=0.036) values for the Schirmer test. In Group 2, there was no statistically significant difference at any time point postoperatively for the Schirmer test. At the same time, there was a statistically significant difference between Group 1 and Group 2 in Week 4 (*P*=0.015). The use of topical Oftassiale was associated with a faster improvement in the signs and symptoms of ocular surface alteration.

**Figure 1:**
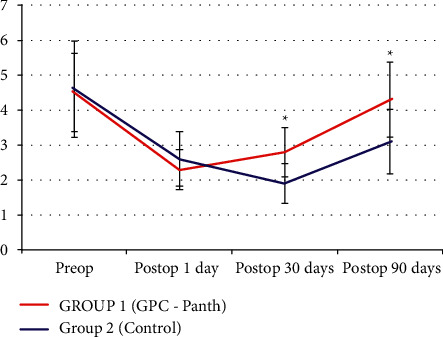
Number of nerve fibers per frames. Compared to baseline, a significant decrease in number of nerve fibers was observed in both treatment groups at postoperative days 1, 30, and 90, with statistically significant between-group differences at 30 and 90 postoperative days.

Compared to the preoperative values in both groups studied, there was a reduced density of the nerve fibers in the postoperative period. But topical Oftassiale treatment was associated with a higher density of nerve fibers at 1 and 3 months than the control group (Figure 1).

Figure 1 also shows that in Group 2, there was a significant postoperative reduction in the number of nerve fibers in the first and third months after surgery.

**Figure 2:**
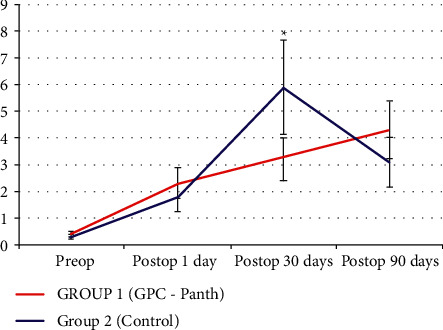
Number of bead-like formations per frames. The subbasal nervous plexus images showed an increase in the number of bead-like formations per millimeter along subbasal nerves in both groups, but it was significantly higher in Group 2 (control) than in Group 1 after one postoperative month.

The number of bead-like formations per millimeter along subbasal nerves was significantly higher in Group 2 (control) than in Group 1 after one postoperative month. Conversely, no significant difference was found between the two groups after three months (Figure 2).

**Figure 3:**
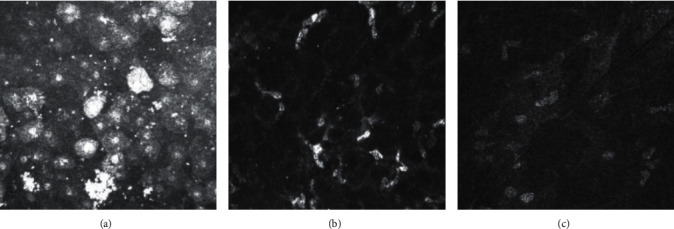
At 30 days after surgery, in both groups, IVCM morphological analysis of the cornea at the incision level showed the alterations at the level of the superficial epithelium with cells with a hyper-reflective nucleus (a), black focal areas between hyper-reflective activated keratocytes and Langerhans cell infiltration (b), and dark stromal striae in the posterior stroma (c).

At 30 days after surgery, in both groups, the morphological analysis of the cornea at the incision level showed the presence of alterations at the level of the superficial epithelium with cells with a hyper-reflective nucleus and moderate stromal edema that still presents hyper-reflective activated keratocytes in the incision area, especially in Group 1 (Figure 3). A significantly higher level of Langerhans cells, detected by IVCM, occurred after 30 days in both groups. The endothelial cells at the level of the cut had normal morphology.

**Figure 4:**
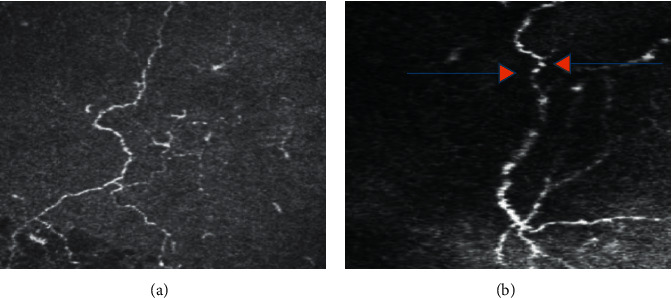
At 30 days after surgery, in both eyes, IVCM images showing a significant reduction in nerve density with an increase in tortuosity and hyper-reflectivity of the fibers (a). The arrows show the presence of number of bead-like formations along the fibers (b).

The subbasal nerve plexus had tortuous and hyper-reflective fibers, especially at one month in both studied groups (Figure 4).

Both treatments were well tolerated, and no clinically significant adverse events were reported.

## 4. Discussion

The cornea is the most densely innervated tissue in the body and receives sensory and autonomic nerve fibers. At the interface between Bowman's layer and the anterior stroma, the stromal nerves form the subepithelial nerve plexus. Cataract surgery induces iatrogenic damage to the corneal nerve structures associated with ocular surface alterations [8].

Cataract surgery temporarily influences the tear film of the ocular surface. Risk factors for deterioration include age, duration of exposure to microscope light, and adequate phacoemulsification time [9].

Most studies show a decrease in TBUT and increased ocular surface staining after cataract surgery, with a resolution of around three months or longer. Tear film thinning and break-up occur mainly due to evaporation from the tear film. TBUT and NIBUT decreased significantly during the first postoperative week and normalized after one to three months [10–12]. Schirmer I test reduced significantly during the first postoperative week and normalized after one to three months. In another study, the TBUT and Schirmer results decreased abruptly one month after surgery and gradually recovered. At the 3-month postoperative time point, the BUT and Schirmer results were still lower than the preoperative level, but they kept rising and finally approached the preoperative level at six months [13–15].

Pre-, intra-, and postoperative risk factors may alter ocular surface homeostasis, resulting in tear film instability, hyperosmolarity, and inflammation of the ocular surface [4, 13, 15]. Consistently, it has been confirmed that phacoemulsification triggers inflammatory stress on eye tissues [16, 17]. In addition, the reduced tear production and increased evaporation increase the osmolarity of the tear film. This event starts an inflammatory process on the ocular surface, leading to the release of multiple cytokines and chemokines, causing cell damage. This cell damage further multiplies the inflammatory processes.

Regarding the ultrastructural corneal changes after cataract surgery, increased infiltration of Langerhans cells in the corneal layer has been reported to be an indicator of postoperative inflammation [18]. In our current analysis, the evidence of Langerhans cells and keratinocyte activation in the stroma may confirm postoperative ocular surface inflammation possibly caused by surgical incision.

After cataract surgery, the osmolarity results increased significantly during the first postoperative week and normalized after three months. Patients with tear osmolarity of 312 mOsm/L or higher are more likely to have more discomfort postoperatively. Moreover, the onset or aggravation of signs and symptoms of dry eye after phacoemulsification, including corneal epitheliopathy and alteration of ocular surface homeostasis, can result in tear film instability, hyperosmolarity, and inflammation [13, 15, 19]. Osmolarity plays an important role in eye health. A strong correlation exists between tear hyperosmolarity and the severity of dry eye syndrome. In patients exhibiting the most severe pathological grade of this condition, tear osmolarity increased to 344 mOsm/L [4]. After cataract surgery, ocular irritation symptoms are very common and may persist for several weeks in some patients [1]. This condition is obviously due to dry eye syndrome postsurgery and is associated with hyperosmotic tears [4].

The present study compared the clinical and morphological results acquired through IVCM between cataract-operated patients undergoing topical adjuvant therapy with Oftassiale and a control group of patients treated with sodium hyaluronate to demonstrate the effects at the level of the corneal incision after cataract surgery. Patients were randomized to receive topical alpha-glycerylphosphorylcholine and D-Panthenol solution or topical hyaluronic acid twice daily six months after surgery.

Glycerylphosphorylcholine (GPC) is the ancestor of “osmo-protective” organic osmolytes. Two complementary mechanisms are recognized for the protective effects of organic osmolytes in cells exposed to hyperosmolarity. First, GPC is associated with a decreasing concentration of intracellular inorganic salts, so compatible organic osmolytes replace inorganic ions. In addition, GPC stabilizes cellular native protein structures and consequently protects them from damage. Moreover, during cellular and tissue repair processes after trauma or corneal surgery, GPC plays an important role in proliferating and migrating epithelial cells (increases membrane fluidity) and plays an important role as an adjuvant and biological modulator of the neuroreparation of the epithelial nerve endings. Integrating the axons in the healing process promotes the synthesis of phospholipids and sphingolipids of the neuronal membranes of the nerve endings [20–22]. Second, Dexpanthenol (Dxp) is a biologically active alcohol-analog of pantothenic acid (PA) converted into PA inside the cell. PA exerts its antioxidative effects by increasing the synthesis of reduced glutathione (GSH) and associated peroxidase enzymes, which serve as the essential protective systems against oxidative stress and lipid peroxidation. Additionally, PA is incorporated into the structure of coenzyme A, stimulates epithelization, and exerts anti-inflammatory effects [23].

In recent years, IVCM has been used more and more to study morphological variations of the ocular surface, as it allows an in vivo microstructural and noninvasive analysis of the human cornea and, consequently, its alterations [5–7]. In this study, the primary outcome was the morphology changes of the corneal tissue adjacent to the surgical incision assessed by IVCM. There is evidence that cataract surgery at the incision level involves a degeneration of the nerve fibers and a consequent density reduction. A recent study demonstrated a significant decrease in nerve fibers one month after surgery until a return to preoperative values eight months after surgery. Surgery has been shown to halve the density of temporal nerve fibers and reduce central ones by 25–35% [18, 24, 25]. Inflammation and irritation of the ocular surface, induced and maintained by dry eye, cause corneal neuropathy expressed by reducing subbasal nerve fiber density.

Alteration in corneal sensitivity is thought to depend on the extent of the corneal incision. A greater incision size corresponds to the slower recovery of corneal sensitivity and wound healing. Corneal sensation dropped centrally and temporally with temporal incision but returned to baseline within one month with a 2.8-mm incision and within three months with a 4.1-mm incision [26, 27]. Mencucci et al. showed that a smaller incision is related to a focal decrease in corneal sensitivity, quicker recovery (1 month), and minimal effect on tear film stability, probably due to the transection of fewer nerve fibers [18].

Although the surgical procedure in cataract surgery is well standardized, surgical experience is an essential factor in constructing the incision [28]. In this study, all incisions were made by the same surgeon and were temporally located.

Our results confirmed other studies showed that mean corneal nerve fiber density, corneal nerve branch density, corneal nerve fiber length, corneal nerve total branch density, corneal nerve fiber area, and corneal nerve fractal dimension significantly decreased one month after the surgery. Significant changes were shown in the central cornea up to 3 months after surgery. The confocal parameters progressively recovered after that (60% at six months and 87% at 8 and 10 months). The temporal plexus fully recovered in all patients at month 8 [6, 29, 30].

The present study has shown that corneal incisions in cataract surgery cause alterations at the level of the nerve fiber layer and, therefore, may be responsible for the symptoms that often accompany the postoperative course. This study also demonstrated that postoperative recovery could be accelerated through the topical use of Oftassiale. The changes in nerve fiber density, highlighted with IVCM, were consistent with the clinical results. The use of Oftassiale could significantly reduce symptoms by improving corneal trophism and accelerating repair mechanisms. Topical therapy with Oftassiale repaired the damaged epithelial barrier and corneal innervation more quickly than the topical treatment.

The epithelial alterations induced by surgery could also determine changes at the level of the corneal stroma, just as the inflammatory process involving the ocular surface can induce apoptosis and increase proteolytic activity in the corneal stroma itself. In addition, the alterations cause proliferation and activation of keratocytes with the secretion of neurogenic growth factors responsible for changes in the number and shape of corneal nerves [31].

The lower number and density of the nerves in the subbasal layer can lead to reduced corneal sensitivity in the group not undergoing therapy with Oftassiale. This reduction may be related to marked corneal hypoesthesia and reduced tear secretion [32]. A more conspicuous number of beads can instead be associated with the high metabolic activity to repair epithelial damage. For example, many of these formations observed in the dry eye could represent an attempt to increase and compensate for abnormal epithelial trophism [5].

The clinical data and the alterations highlighted by the IVCM in the context of our study propose a better picture of what concerns the group treated with Oftassiale than the untreated group.

In conclusion, this study confirmed that IVCM helps assess the morphological changes at the corneal incision tunnel after cataract surgery due to its ability to provide microscopic details in vivo. Topical therapy with alpha-glycerylphosphorylcholine and D-Panthenol eye drops in patients undergoing cataract surgery promoted and stabilized the reepithelialization process. This fixed combination also accelerated and modulated the repair of the corneal innervation.

## Data Availability

Data are available on request.
